# Relationship between prediction accuracy and uncertainty in compound potency prediction using deep neural networks and control models

**DOI:** 10.1038/s41598-024-57135-6

**Published:** 2024-03-19

**Authors:** Jannik P. Roth, Jürgen Bajorath

**Affiliations:** Department of Life Science Informatics and Data Science, B-IT, LIMES Program Unit Chemical Biology and Medicinal Chemistry, Rheinische Friedrich-Wilhelms-Universität, Friedrich-Hirzebruch-Allee 5/6, 53115 Bonn, Germany

**Keywords:** Uncertainty quantification, Machine learning, Compound potency prediction, Prediction accuracy, Cheminformatics, Cheminformatics

## Abstract

The assessment of prediction variance or uncertainty contributes to the evaluation of machine learning models. In molecular machine learning, uncertainty quantification is an evolving area of research where currently no standard approaches or general guidelines are available. We have carried out a detailed analysis of deep neural network variants and simple control models for compound potency prediction to study relationships between prediction accuracy and uncertainty. For comparably accurate predictions obtained with models of different complexity, highly variable prediction uncertainties were detected using different metrics. Furthermore, a strong dependence of prediction characteristics and uncertainties on potency levels of test compounds was observed, often leading to over- or under-confident model decisions with respect to the expected variance of predictions. Moreover, neural network models responded very differently to training set modifications. Taken together, our findings indicate that there is only little, if any correlation between compound potency prediction accuracy and uncertainty, especially for deep neural network models, when predictions are assessed on the basis of currently used metrics for uncertainty quantification.

## Introduction

Predictions of most machine learning (ML) models including all deep learning models^1^ cannot be rationalized via human reasoning, which is often referred to as the “black box” nature of such models^2^. Hence, as the use of ML is increasing in many areas of science, including pharmaceutical research^3,4^, there also is increasing interest methods for ML model explanation^5–7^. In pharmaceutical research, the prediction of various molecular properties, in particular, biological activity, is a primary application of standard ML and deep learning^4,8^ models and a major focal point of approaches for model explanation^6^. Methods for explaining ML predictions are complemented by approaches for assessing the confidence or uncertainty with which a model reaches predictions. First and foremost, “uncertainty quantification” (UQ) of predictions has gained popularity, especially for deep learning models^9,10^. For instance, UQ helps to better understand inconsistent model performance across different application domains and aids in the selection of appropriate metrics for evaluating model performance^9^. In medicinal chemistry, deep neural network models are increasingly used for different applications, even though their performance is often at best on par with simpler ML models, for instance, in molecular property prediction^11^. This is at least in part due to the situation that medicinal chemistry data sets are typically limited in size, making it difficult for data-hungry deep learning techniques to reach high performance levels^12^. For computationally complex models, UQ techniques such as dropout, deep ensembles, or mean–variance-estimation can be employed^13,14^. However, the performance of UQ methods also depends on data set features and model architectures^15,16^, making it often complicated to assess ML model performance in a consistent manner, especially when the performance itself displays substantial data set dependence. Some ML approaches yield uncertainty estimates. For example, Gaussian process modeling includes calibrated uncertainties, but is not widely applied in pharmaceutical research, due its vulnerability to high-dimensional molecular (descriptor) representations that are commonly used in chemoinformatics and medicinal chemistry^12,17^. For ML methods such as random forest (RF) and support vector machine (SVM) or k-nearest neighbor (kNN) models, which are mainstays in chemoinformatics, a variety of model-specific UQ techniques has been introduced^18–23^. However, the use of model-agonistic UQ estimates is generally preferred to enable direct comparisons of predictions made by different types of ML models. Thus far, generally applicable model ensemble-based techniques have predominantly been used for UQ in compound property predictions^15,24^. To this end, alternative UQ metrics can be employed that conceptually differ and rely on specific assumptions^25,26^. For example, the frequently used negative log-likelihood (NLL) or miscalibration area metrics assume an underlying distribution of prediction errors.

In this work, we have analyzed prediction uncertainty of deep neural networks and simple control methods in compound potency prediction and explored relationships between prediction accuracy and uncertainty. For the assessment of prediction variance and model confidence, different performance measures and alternative models were investigated.

## Methods

### Compounds and activity data

Compounds and activity measurements were extracted from ChEMBL (version 33)^27^. Compounds with a molecular mass of less than 1000 Da, an assay confidence score of 9, and a numerically specified potency (IC_50_) value were selected. Only assays with single proteins were considered. Compounds with a potency of less than 10 μM or more than 10 pM were disregarded. If multiple potency values were reported for the same compound-target pair, the values were averaged and only retained if the potency values fell into the same order of magnitude (tenfold). Undesired pharmaceutical targets such as anti-targets (hERG, cytochrome P450, P-glycoprotein, albumin, UDP-glucuronosyltransferase, glutathione S-transferase, N-acetyltransferase, or sulfotransferases), were removed prior to filtering for potential assay interference compounds (activity artifacts) using publicly available filters including filters for pan assay interference compounds (PAINS)^28^, as implemented in RDKit^29^, Eli Lilly Medicinal Chemistry Rules^30^, and potential aggregators^31^. Only human targets were considered. A total of 21 curated compound activity classes with more than 1000 qualifying compounds and diverse (functionally distinct) pharmaceutical targets were selected, as reported in Table 1, representing some of the largest high-quality activity classes that are currently available. All compounds were represented using folded Morgan fingerprints^32^ with a length of 2048 bits and a bond radius of 2. The fingerprints were generated using RDKit^29^.

**Table 1:** Activity classes.

ChEMBL target ID	Compounds	Target
279	2567	Vascular endothelial growth factor receptor 2
220	2337	Acetylcholinesterase
325	2169	Histone deacetylase 1
203	1695	Epidermal growth factor receptor erbB1
1914	1679	Butyrylcholinesterase
4005	1640	PI3-kinase p110-alpha subunit
1865	1565	Histone deacetylase 6
260	1555	MAP kinase p38 alpha
230	1531	Cyclooxygenase-2
2409	1474	Epoxide hydratase
2039	1473	Monoamine oxidase B
284	1437	Dipeptidyl peptidase IV
4822	1373	Beta-secretase 1
3130	1358	PI3-kinase p110-delta subunit
3717	1328	Hepatocyte growth factor receptor
1,163,125	1191	Bromodomain-containing protein 4
3267	1188	PI3-kinase p110-gamma subunit
4296	1178	Sodium channel protein type IX alpha subunit
262	1178	Glycogen synthase kinase-3 beta
2971	1074	Tyrosine-protein kinase JAK2
333	1005	Matrix metalloproteinase-2

For each activity class, the ChEMBL target ID, number of compounds, and target name are provided.

### Training data modification

To explore potential effects of different training data distributions on the performance of UQ methods, the activity data were modified in different ways.

#### Balanced data

The training set was split into three potency bins: pIC_50_ ≤ 5.5, 5.5 < pIC_50_ ≤ 7.5, and pIC_50_ > 7.5. The data was balanced by counting the number of samples in each of these three bins. The number of samples in the smallest bin determined how many compounds were randomly selected from the other two larger bins (minority sampling). This procedure generated training sets in which the number of compounds per potency bin was identical.

#### Reduced data

A training set was split into three bins, as described above. Compounds in the central bin were removed from the training set. Since most compounds in activity classes from medicinal chemistry are active in the micromolar range, corresponding to the central bin, this data reduction made it possible to study the effects of removing the compounds in the most populated potency sub-range on the performance of UQ methods.

### Models

For potency prediction, a variety of regression models were derived. For each activity class, 10 random compound splits (70% training data, 30% test data) were carried out. For each split, hyperparameter optimization was performed for ML models using training data. Regression model performance was evaluated using the mean squared error (MSE) and the coefficient of determination (R^2^), which are commonly used metrics for regression tasks across different applications domains.

#### Ensembles of machine learning models

Ensembles of kNN and decision tree (DT) models were implemented using scikit-learn^33^. DT ensembles represent RF models. The following hyperparameters were optimized for all ensembles using *tune*^34^: max_samples (from 0.05 to 1.00), max_features (from 0.05 to 1.00) and n_estimators (100, 150, or 200). For kNN models, the number of neighbors (1, 3, or 5) was optimized. Tanimoto distances^35^ were used for the kNN models. Five-fold internal cross-validation was carried out during optimization and the MSE was used as the loss function.

#### Feed-forward neural network with dropout

Feed-forward neural networks (FFNNs) were implemented using PyTorch^36^. Two different architectures introduced previously^10,16^ were employed including FFNNs consisting of four hidden layers with sizes 1000, 1000, 100, 10, and the final output layer of size 1 (termed FFNN large). In addition, FFNNs with two hidden layers of size 300 were generated (FFNN small). The ReLU activation function was used in all hidden layers. The Adam optimizer with a learning rate of 1 × 10^–3^ was employed^37^. A batch size of 32 and a total of 600 epochs were used. Dropout layers with rate of 10%, 20%, or 50% were added between all hidden layers. The MSE was used as the loss function. For each model, 100 prediction trials were carried out to generate statistically sound predictions.

#### Mean–variance estimation

Mean–variance estimation networks (MVEs) were also implemented using PyTorch^36^. Two different architectures were employed^10^ including MVEs consisting of four hidden layers with sizes 1000, 1000, 100, 10 and the final two output layers of size 1 (MVE large). In addition, MVEs with two hidden layers of size 300 were generated (MVE small). The ReLU activation function was used on all hidden layers and the Adam optimizer^37^ with a learning rate of 1 × 10^–3^. A batch size of 32 was applied and the model was trained for a maximum of 4000 epochs using an early termination criterion if the error did not decrease over 100 epochs. As a loss function, NLL assuming a normal distribution was used.

#### Single machine learning models

For comparison with neural networks, basic ML models were implemented using scikit-learn^33^. A single DT and kNN were used. Within each split of the kNN models, hyperparameter optimization of the number of neighbors (1, 3, or 5) was carried out using tune^34^ with five-fold internal cross-validation was performed. The MSE was used as a loss function.

### Metrics

#### Negative log likelihood

NLL is a widely applied score in ML^38,39^. For a regression task assuming a normal distribution, the NLL is calculated as,

$${\text{NLL}}\left(D\right)=\frac{1}{2\left|D\right|}{\sum }_{i}^{\left|D\right|}{\text{ln}}\left(2\pi \right)+{\text{ln}}\left({\sigma }_{i}^{2}\right)+\frac{{\left({\widehat{y}}_{i}-{y}_{i}\right)}^{2}}{{\sigma }_{i}^{2}},$$

where D is the data set containing |D| samples. NLL provides a balance between the actual error of the prediction and the corresponding estimated uncertainty.

#### Miscalibration area

The miscalibration area quantifies how well predicted uncertainties are calibrated with respect to an underlying distribution^40^. This is accomplished by comparing the fraction of compounds falling within *x* standard deviations of the mean and the expected fraction based on an assumed distribution with the variance equal to the predicted uncertainty. As an example, assuming a normal distribution, one expects 68% of the predicted samples to fall within one standard deviation. The miscalibration area calculates the difference between the theoretical and observed ratio and for multiple instances of *x* and integrates over the entire data range. Thus, the miscalibration area measures if a model is systematically over- or underconfident. A miscalibration area of zero indicates a well-calibrated model.

Notably, this metric has a potential shortcoming. As a consequence of the integration, over- an underconfident predictions might cancel out, leading to an overall miscalibration area close to zero, although a model might not be well calibrated^10^. Therefore, the absolute miscalibration area was implemented, which sums the absolute values of deviations over the entire data set. Consequently, the absolute miscalibration area fully accounts for deviations but no longer indicates whether a model might be model is over- or underconfident. By comparison with the original miscalibration area value, potential error cancellation effects can be identified. In the following, the miscalibration area is denoted ‘A’ and the absolute miscalibration area ‘A_abs_’ (note that the absolute value of ‘A’ is not equal to ‘A_abs_’). The absolute miscalibration area is related to the calibration error that calculates the squared deviation from the assumed distribution^41^.

#### Spearman’s rank correlation coefficient

Spearman's rank correlation coefficient ρ quantifies the correlation of rankings for two different variables. Its use is motivated by the notion that predictions with large uncertainties should also have larger deviations from the true value. For two vectors v_1_ and v_2_, we denote their rankings as r_v1_ and r_v2_, respectively. The Spearman rank correlation coefficient ρ is calculated as

$$\rho \left({v}_{1}{\text{,v}}_{2}\right)=\frac{{\text{cov}}\left({r}_{v1}{\text{,r}}_{v2}\right)}{{\text{std}}\left({r}_{v1}\right){\text{std}}\left({r}_{v2}\right)},$$

where cov is the covariance and std the standard deviation of the respective vector rankings. Notably, any monotonically increasing function applied to these vectors retains the value of ρ. The Spearman’s rank correlation coefficient is calculated to quantify potential correlation between the squared prediction error and the predicted uncertainty.

## Results

### Performance of machine learning models

**Figure 1:**
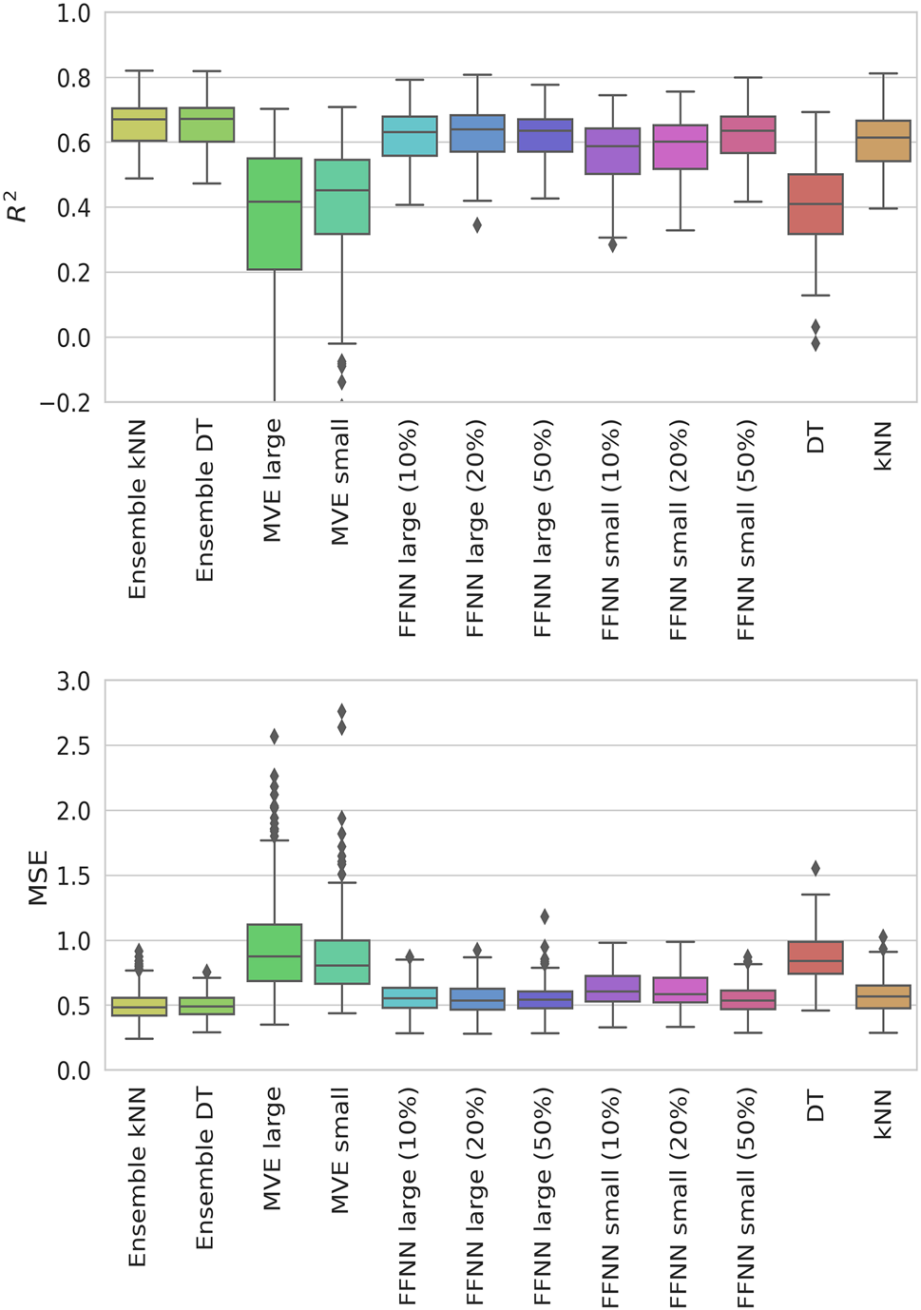
Performance of different approaches for compound potency prediction. The prediction accuracy of kNN and DT ensembles, MVE small, MVE large, FFNN small, FFNN large, with different dropout rates (%), a single DT, and kNN is evaluated on the basis of R^2^ (top) and MSE values (bottom). Results are reported for all activity classes.

Initially, the performance of the different ML models described above was determined across all activity classes in Table 1 and compared. Boxplots in Fig. 1 summarize compound potency predictions over independent trials on the basis of R^2^ and MSE values respectively.

The models produced overall accurate, stable, and closely corresponding predictions with median MSE values ~ 0.6 and R^2^ values of ~ 0.6–0.7, with the exception of MVE models and the single DT model that were less accurate, albeit by only a small margin, with median MSE values of ~ 0.8–0.9 and R^2^ values of ~ 0.4–0.5. By contrast, kNN, the simplest of all approaches, essentially met the prediction accuracy of the complex FFNN models, consistent with earlier findings for systematic compound potency predictions over a wide range of activity classes^11,42^. Different FFNN versions displayed very similar performance. While single kNN and kNN ensembles displayed only minor differences, with median MSE values of 0.58 ± 0.14 to 0.50 ± 0.12, respectively, the DT ensemble (0.50 ± 0.10) improved the accuracy of a single DT (0.87 ± 0.18). However, performance differences between all models were confined to small median R^2^ and MSN intervals of ~ 0.3 and ~ 0.4, respectively. The generally narrow value distributions in Fig. 1 (except for MVE) also indicated very similar predictive performance across the different activity classes.

### Uncertainty quantification

**Figure 2:**
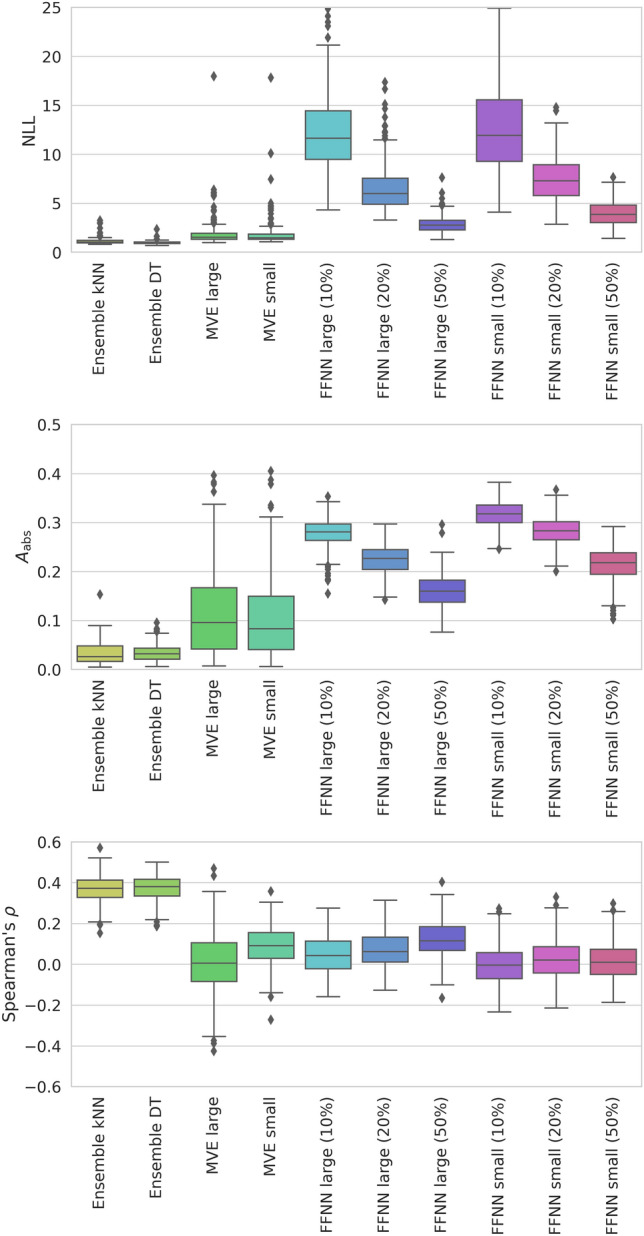
Uncertainty quantification for different models. For the predictions reported in Fig. 1, UQ is carried out using NLL (top), A_abs_ (middle) and ρ (bottom) are shown. Results are reported for all activity classes.

Next, UQ was carried out for all predictions. Figure 2 shows the results obtained on the basis of NLL, A_abs_, and *ρ* values for the different models. For NLL and A_abs_, a normal distribution of prediction errors is assumed. NLL balances prediction errors with corresponding prediction uncertainties (a low value indicates small prediction errors and uncertainties) while A_abs_ measures differences between the actual error distribution and the assumed normal distribution (a value of 0 means that there is no difference).

For UQ, different trends were observed in the presence of overall closely corresponding prediction accuracies. For NLL, most values of the kNN and DT ensembles were close to 0 (with very narrow value distributions). Similarly low NLL values were obtained for MVEs (with some statistical outliers). By contrast, larger values were obtained for FFNNs (small and large) with a dropout rate of 50%, which further increased significantly with decreasing dropout rates (with largest median values exceeding 10). For A_abs_, closely corresponding trends were observed, except that value distributions for MVEs were in this case much broader than for FFNNs. Given that the maximal value of A_abs_ is 0.5, some large deviations were detected (with median values of ~ 0.3). Thus, on the basis of both metrics, comparably accurate predictions using different methods displayed in part large differences in prediction uncertainty, especially for FFNN variants with varying dropout rates.

Rank correlation coefficient *ρ* was calculated to measure the correlation between the observed prediction errors (R^2^) and the predicted variance. For kNN and DT ensembles, limited correlation with median *ρ* values of ~ 0.4 was detected. By contrast, for neural network methods, values close to 0 were obtained, indicating the absence of rank correlation between actual prediction errors and estimated uncertainties, consistent with the findings discussed above.

### Potency interval dependence of predictions

**Figure 3:**
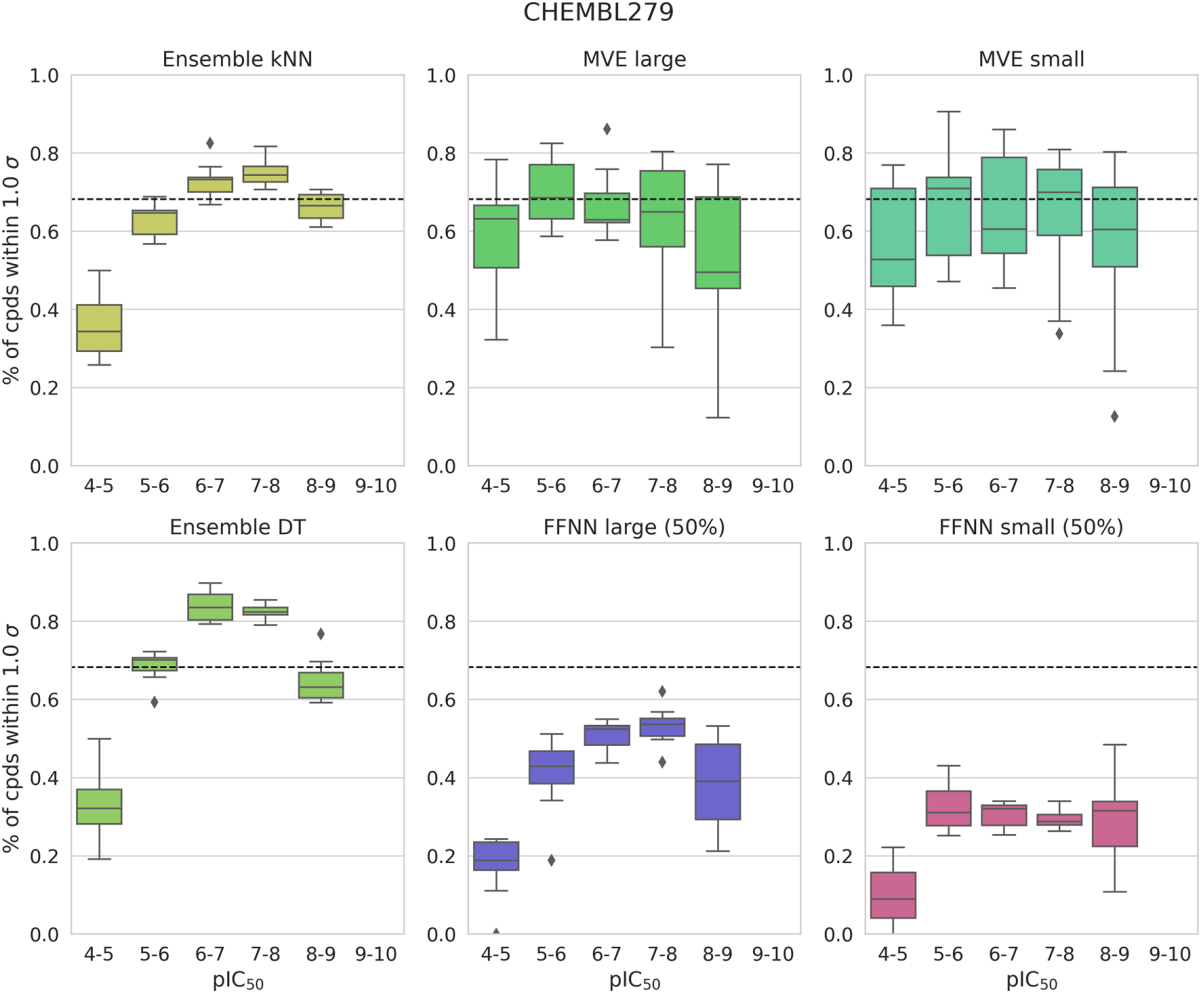
Potency interval-dependent model performance. For activity class 279, predictions of selected models were monitored for test compounds falling into different potency intervals. The dashed line indicates the theoretical ratio of compounds within one standard deviations of the mean assuming a normal distribution of prediction uncertainties. The ratio of compounds predicted to be within 1.0 σ by a model is plotted for different potency bins.

As an alternative to applying the NLL and A_abs_ metrics, we also assessed the confidence of a predictive model by assuming a normal distribution of prediction uncertainties and monitoring the proportions of compounds falling within one standard deviation of the mean. For this analysis, test compounds were assigned to different potency intervals (bins) spanning the logarithmic potency range from 4 to 10, and the potency predictions reported in Fig. 1 were separately monitored for test compounds falling into each bin. As already indicated by the narrow value distributions for the different activity classes in Fig. 1, we found that the results of potency interval-based analysis of the predictions were closely corresponding for the different activity classes. Therefore, in Fig. 3, representative results are presented for activity class 279 (Table 1) and additional examples are shown in Supplementary Fig. S1.

In the presence of a normal distribution, 68.27% of the predicted test compounds are expected to fall within 1.0 σ. Figure 3 shows the observed ratios for test compounds in each potency bin. If the ratio was smaller for a given model than the expected one (68.27%), the model was considered “over-confident” because it under-predicted uncertainties; if the observed ratio was larger, the model was considered “under-confident” because it over-predicted uncertainties relative to the normal distribution. Figure 3 clearly shows that model confidence varied across compound potency ranges. Specifically, for kNN and DT ensembles, which yielded overall only small A_abs_ values (Fig. 2), the models tended to be over-confident, especially for weakly potent, but also highly potent compounds. By contrast, the models tended to be under-confident for compounds with intermediate potency (representing the largest fraction of compounds per data set). Hence, the overall small A_abs_ values primarily resulted from averaging over the entire test sets. MVE and FFNN models yielded similar potency interval-dependent distribution patterns, but at different compound proportion levels. MVE models exhibited much broader value distributions (larger variance) than the ensembles and tended to be slightly over-confident across the entire potency range (in the presence of largest prediction errors, Fig. 1). Moreover, FFNN models had smaller variance than MVE models, but were strongly over-confident across the entire potency range. Thus, monitoring predictions for test compounds in different potency intervals revealed heterogeneity of model confidence levels (Fig. 3) in the presence of different UQ characteristics (Fig. 2).

**Figure 4:**
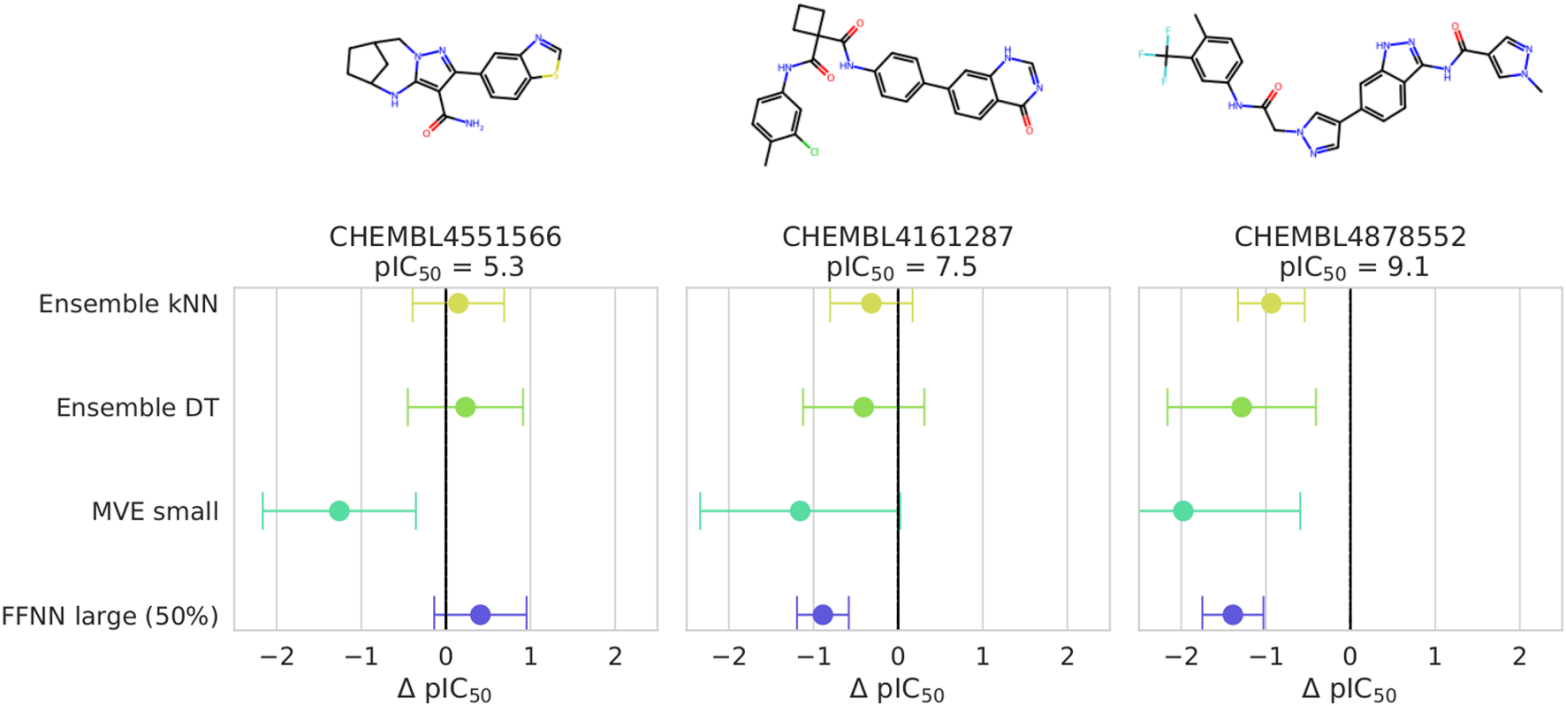
Prediction error vs. uncertainty. For activity class 279, the prediction error (x-axis) is reported for ensemble of kNNs and DTs, the MVE small, and the FFNN large with 50% dropout rate. Prediction uncertainty is indicated by error bars with a width of ± 1.0 standard deviations. Three exemplary test compounds were selected from low, intermediate, and high potency intervals (from the left to the right).

Figure 4 compares the prediction errors and associated uncertainties for different models derived using the same training set and reveals consequences of the potency interval-dependent heterogeneity for exemplary predictions of three test compounds. Here, the best performing MVE and FFNN variants (Fig. 1) were selected (i.e. MVE small and FFNN large with 50% dropout, respectively). Although the prediction uncertainties were similar for each model and test compound (except for one instance of the FFNN model), the prediction errors increased for increasing compound potency (where potency values were consistently underpredicted). Notably, none of the models predicted the potency of the highly potent compound with reasonably accuracy, yielding prediction errors of one to two orders of magnitude. The difference in calibration quality across the potency range is analogous to results obtained for prediction quality^42^. The effect of the data distribution is analyzed in detail below.

### Calibration dependence on training data

**Figure 5:**
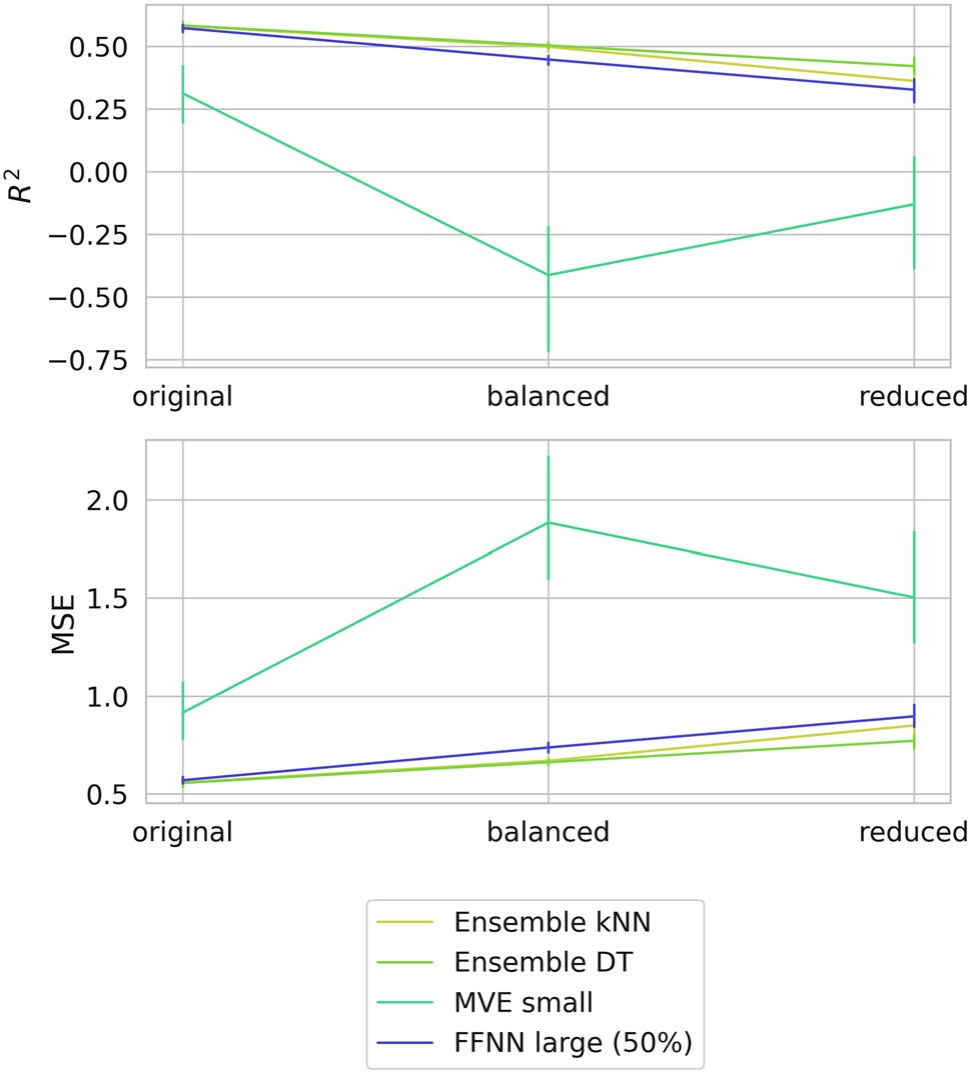
Performance of original and re-calibrated models. For activity class 279, the performance of selected models derived on the basis of modified training sets is assessed on the basis R^2^ (top) and MSE values (bottom).

**Figure 6:**
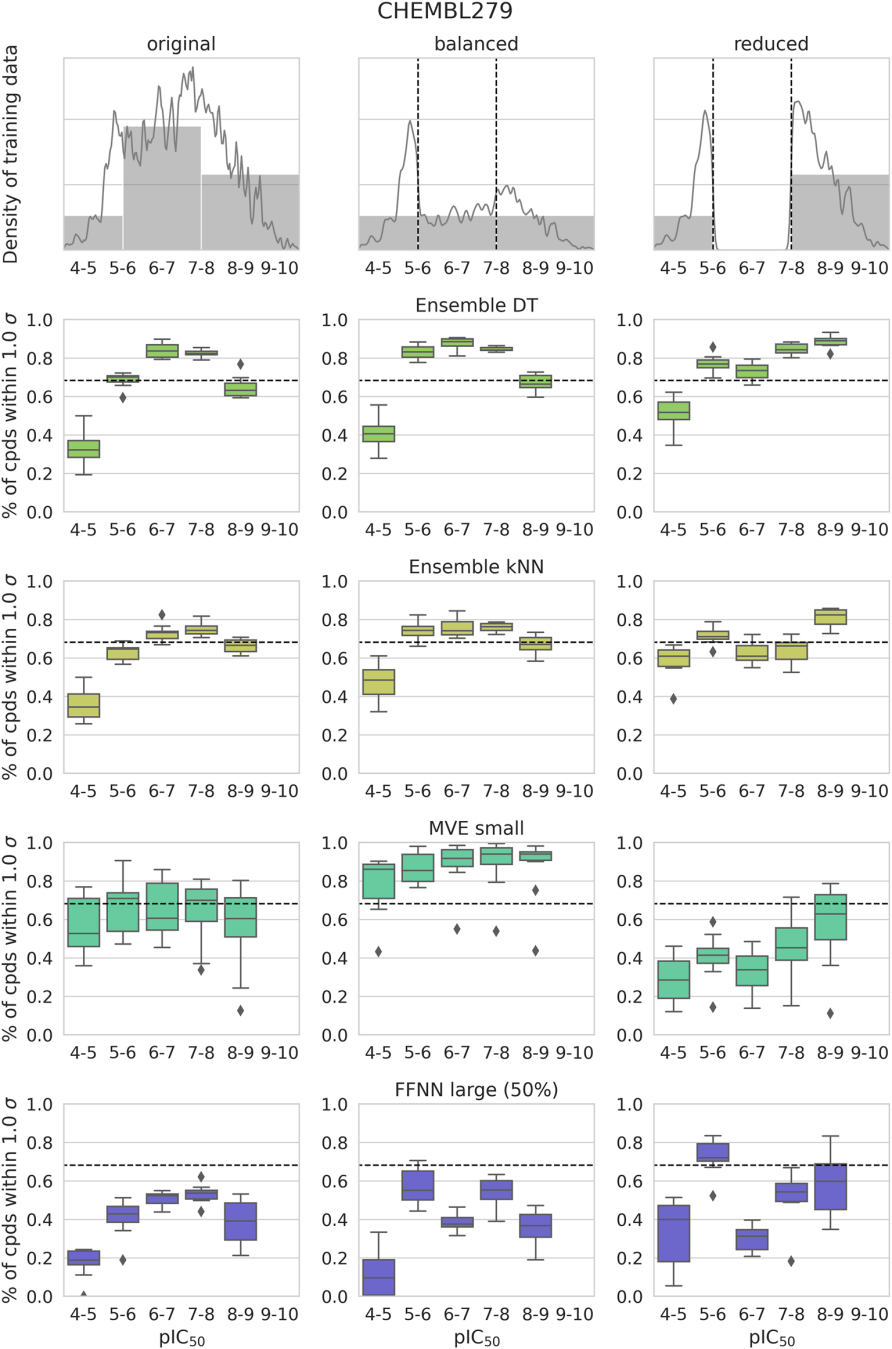
Training data dependence. For activity class 279, selected models were derived on the basis of different training sets (original, balanced, reduced) and calibrated. The graphs at the top report the compound density for different training sets. The potency bin-based data presentation is according to Fig. 3.

In light of the findings discussed above, we re-trained all models for two training set variants, in which the compound population was balanced across all potency bins or reduced by omitting training compounds falling into the intermediate potency sub-range 5–7. Then, test predictions and model confidence assessment were repeated for re-calibrated models. Again, closely corresponding results were obtained for the different activity classes. Figure 5 compares the predictive performance of the original and re-calibrated models for class 279 and Fig. 6 reports the confidence analysis for this class (additional examples are shown in Supplementary Fig. S2). At the top of Fig. 6, training compound density is shown for the original training set (with predominance of compounds having intermediate potency), the balanced, and reduced set.

Figure 5 reveals different changes in the performance of models derived on the basis of modified training sets. For kNN and DT ensembles and the FFNN model, prediction errors slightly increased for original over balanced to reduced training data sets, indicating that smallest global errors were largely determined by test compounds with intermediate potency. Since compounds with intermediate potency were not contained in reduced training sets, prediction errors of the resulting models were largest on a relative scale. Nonetheless, even these models only yielded limited prediction errors (within one order of magnitude). By contrast, the MVE model departed from the prediction characteristics of the others. Here, changes as a consequence of training set modifications were much larger, with R^2^ values close to 0 for balanced and reduced training sets and overall largest prediction errors for the model derived on balanced data, reflecting non-expected model behavior.

Figure 6 compares the potency interval-dependent characteristics of models derived based on the original (Fig. 3) and modified training sets. For balanced training sets, kNN and DT ensembles displayed only small alterations in model confidence relative to the original models. Hence, compound balancing across different potency intervals did not eliminate or reduce the interval-dependent heterogeneity discussed above. Surprisingly, however, models derived from reduced data sets essentially retained expected uncertainties in intermediate potency intervals although training compounds from these intervals were not available. These findings indicated that calibration for ensemble methods was largely a consequence of the global potency value distribution and the resulting mean, rather than the predominant population of intermediate potency ranges with training compounds; an interesting finding. Notably, for ensemble kNN, the model derived from the reduced training set closely matched the expected model variance.

For the neural network models, different effects were observed as a consequence of training set modification. While the original MVE models were consistently over-confident, balanced training sets produced consistently under-confident models, whereas reduced training sets regenerated over-confident models. These model characteristics were consistent with the training set-dependent changes in predictive performance discussed above. The FFNN models displayed varying potency interval-dependent distribution patterns as a consequence of training set modifications, but remained over-confident (with only one exception for reduced training data). Hence, FFNNs were overall more stable than MVE models.

## Conclusion

In this work, we have investigated machine learning models of very different complexity for compound potency predictions to explore relationships between prediction errors and the uncertainty of predictions. The assessment of prediction uncertainty or model confidence complements approaches for model explanation and aids in rationalizing predictions. Consistent with earlier findings, models of different complexity including neural network variants and DT or kNN (ensembles) produced reasonable predictions of mostly comparable accuracy. However, for these predictions, different uncertainties were detected on the basis of commonly used metrics such as NLL or the miscalibration area. For simple kNN and DT ensembles, there was detectable correlation between prediction errors and the predicted variance, but for neural network models, no correlation was observed. These observations might in part be attributable to approximations underlying commonly used UQ metrics assuming normal data distributions. However, there also were a number of unexpected findings contributing to substantial variations in prediction uncertainty such as the strong dependence of uncertainties on the dropout rates of FFNN models or substantial performance variations of MVE models based on modified training sets. Moreover, we detected a generally strong potency interval dependence of model confidence, giving rise to highly variable value distribution patterns for different methods and over- or under-confident model decisions across different potency intervals. This behavior might also be affected by the limited dynamic range of the data sets, which represents an inherent property of potency prediction tasks. Additionally, underlying data structures such as analogue series have might have an effect on the interplay between prediction performance and uncertainty quantification. On a similar vein, ensemble and neural network models responded rather differently to training set modifications. Surprisingly, however, test compounds with intermediate potency that generally dominated the composition of activity classes were still predicted with reasonable accuracy if such compounds were excluded from model derivation. These observations indicated that predictions of ML models (except the kNN control) were mostly guided by global data distributions and resulting median potency values, in the presence of varying prediction uncertainty. This was also consistent with the in part large errors observed when predicting highly potent compounds. Taken together, the findings reported herein revealed an unexpectedly complex interplay between the performance of neural network and control models and their prediction uncertainties. In light of these findings, care should be taken in the assessment and interpretation of model uncertainty or confidence. Given the results of our analysis, future research might focus, for example, on the design and evaluation of further advanced UQ metrics or alternative analysis schemes for assessing prediction variance of deep learning models.

As suggested by one of the reviewers, we also note that predictions using deep neural networks and other ML methods incorporating uncertainty quantification are also carried out in other pharmaceutically relevant research fields that are distantly or unrelated to the topic of our study. For instance, graph neural networks have been applied for the prediction of compound cardiotoxicity^43^. For drug development, assessing potential risks and adverse effects associated with candidate compounds as early as possible is highly desirable. The addition of uncertainty quantification methods in ML can guide risk assessment and accelerate experimental evaluation. Similar models are applicable to predict the association metabolites and disease states^44^. In such application areas, uncertainty quantification supports the understanding of applied models based on human reasoning, helps to judge the anticipated reliability of predictions for practical applications, and thereby increases the acceptance of models in interdisciplinary research settings. The benefits of uncertainty quantification also extend to other areas in pharmaceutical and biological research, for example, for time series-based predictions of cell death in different biological systems using ML models^45–47^. Here, evaluating the uncertainty of different models can aid in prioritizing alternative ML approaches and in advancing the understanding of suitable application domains and domain-dependent model limitations. Such applications illustrate the potential of uncertainty quantification in complementing predictions using deep neural networks and other ML methods in different fields.

### Supplementary Information

Supplementary Figures.

## Data Availability

Calculations were carried out using publicly available software and compound data.

## Code availability

Code used for the generation of the models, performance analysis, and generation of figures is freely available via the following link: https://doi.org/10.5281/zenodo.10825202.
