# Supplementary material for: Relationship between prediction accuracy and uncertainty in compound potency prediction using deep neural networks and control models
Source: Sci Rep. 2024 Mar 19;14:6536. doi: 10.1038/s41598-024-57135-6 (PMC10950896; doi:10.1038/s41598-024-57135-6)

Supplementary Figures

**Relationship between prediction accuracy and uncertainty in compound potency prediction using deep neural networks and control models**

Jannik P. Roth and Jürgen Bajorath

Department of Life Science Informatics and Data Science, B-IT, LIMES Program Unit Chemical Biology and Medicinal Chemistry, Rheinische Friedrich-Wilhelms-Universität, Friedrich-Hirzebruch-Allee 5/6, D-53115 Bonn, Germany.

**Figure legends**

Supplementary Figure S1. Potency interval-dependent model performance. For activity class (a) 220, (b) 325, and (c) 203 (Table 1), predictions of selected models were monitored for test compounds falling into different potency intervals. The dashed line indicates the theoretical ratio of compounds within one standard deviations of the mean assuming a normal distribution of prediction uncertainties. The ratio of compounds predicted to be within 1.0 σ by a model is plotted for different potency bins.

Supplementary Figure S2. Training data dependence. For activity class (a) 220, (b) 325, and (c) 203, selected models were derived on the basis of different training sets (original, balanced, reduced) and calibrated. The graphs at the top report the compound density for different training sets. The potency bin-based data presentation is according to Supplementary Fig. S1.

**Fig. S1a**


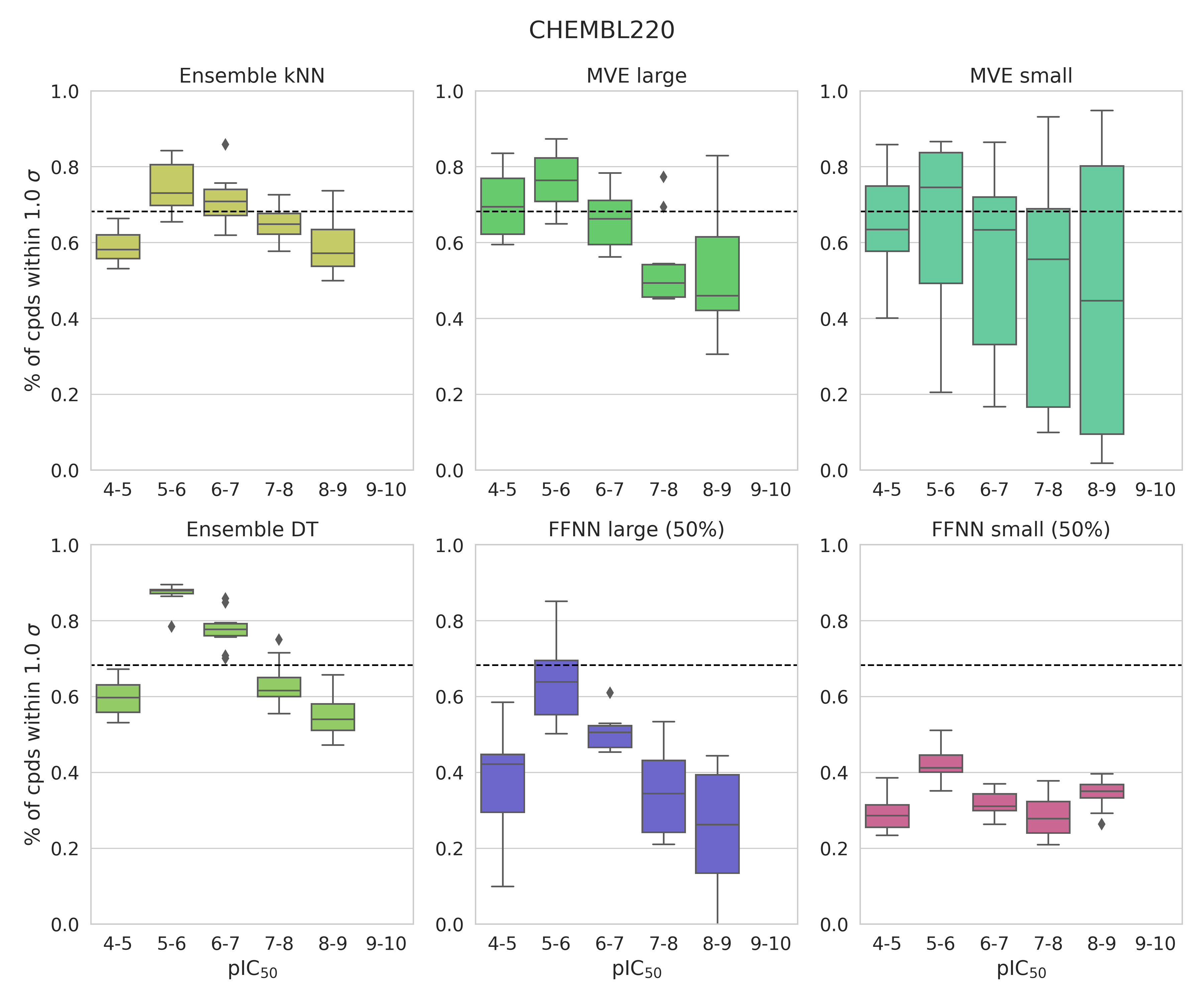


**Fig. S1b**

**
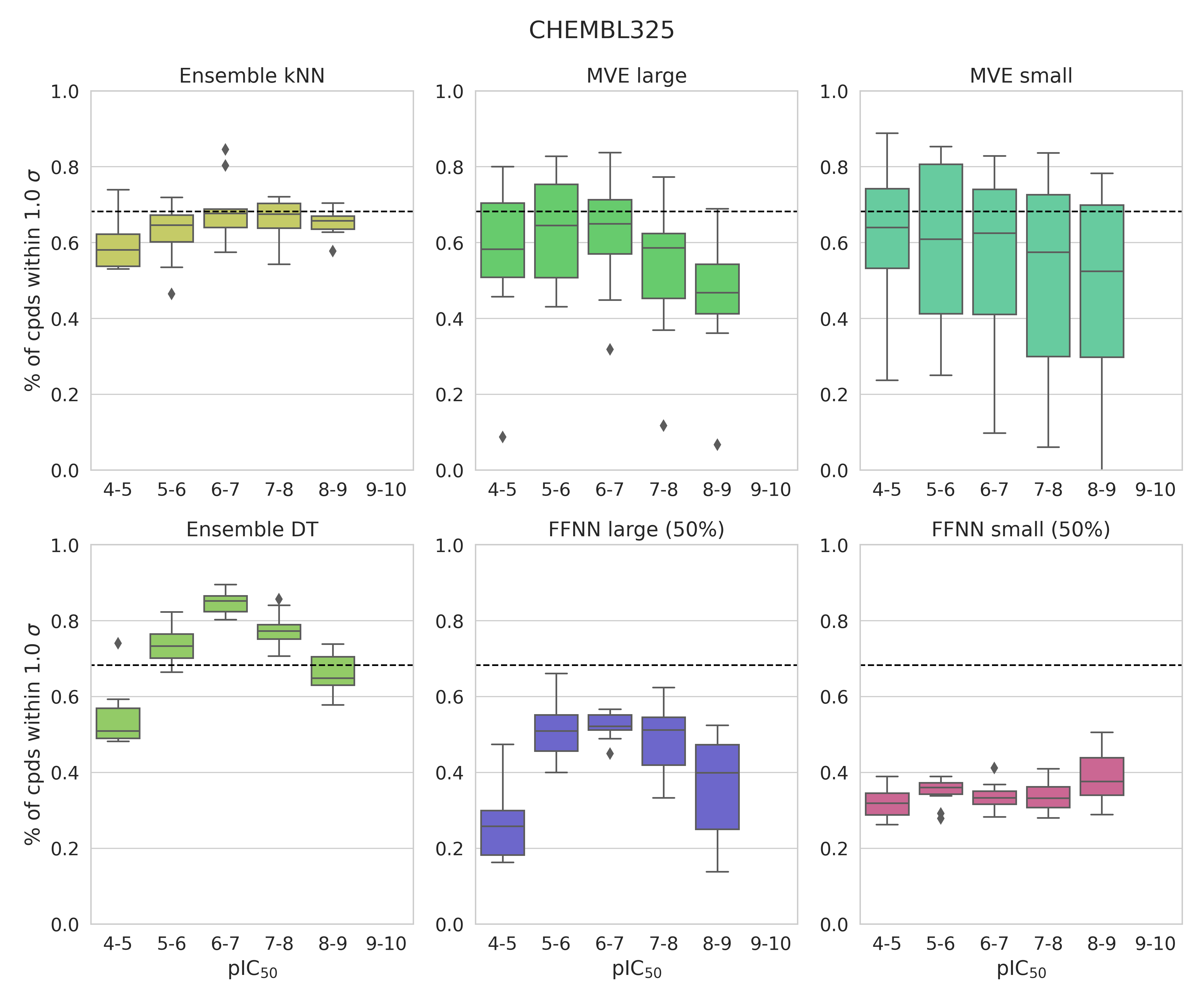
**

**Fig. S1c**

**
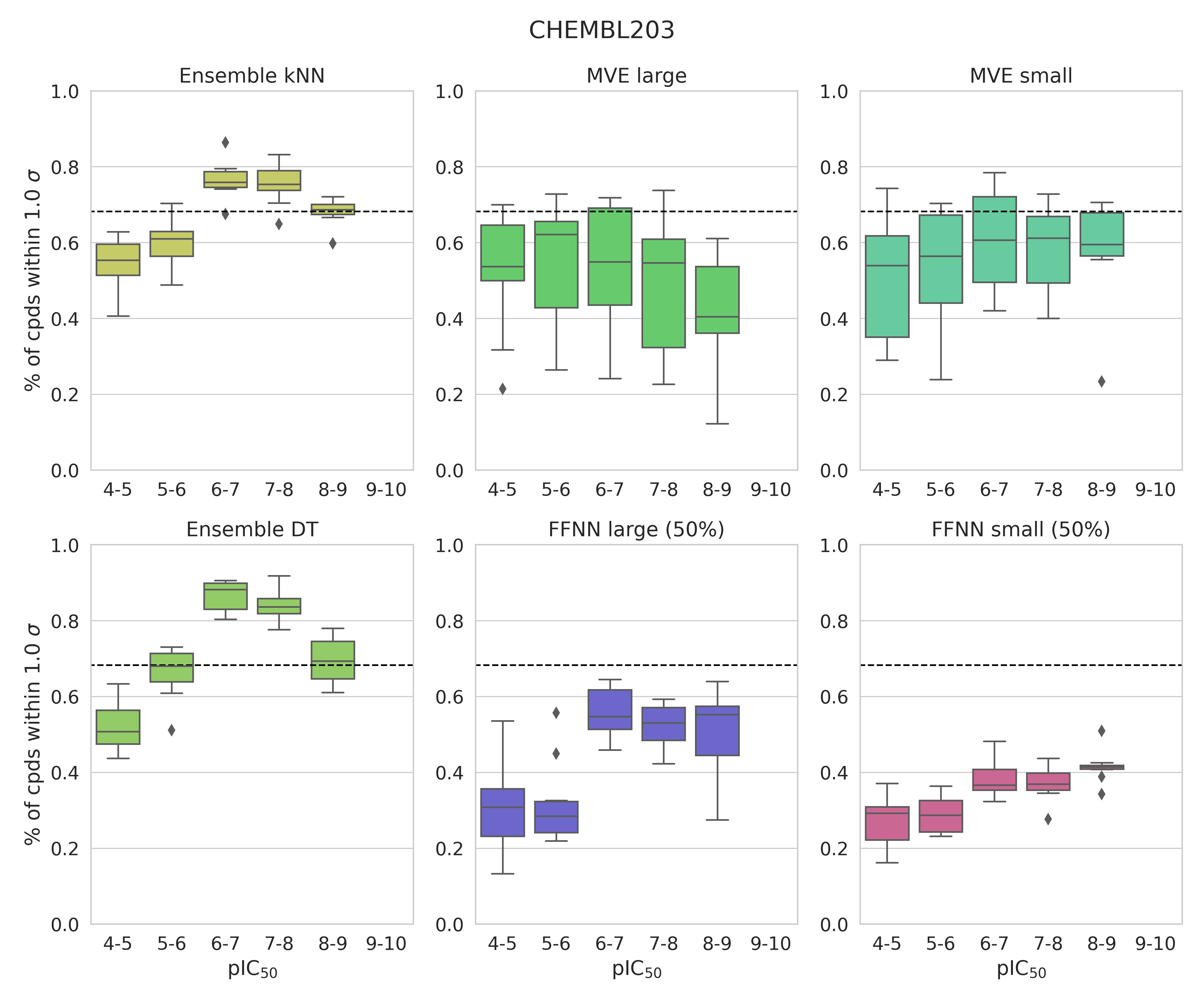
**

**Fig. S2a**


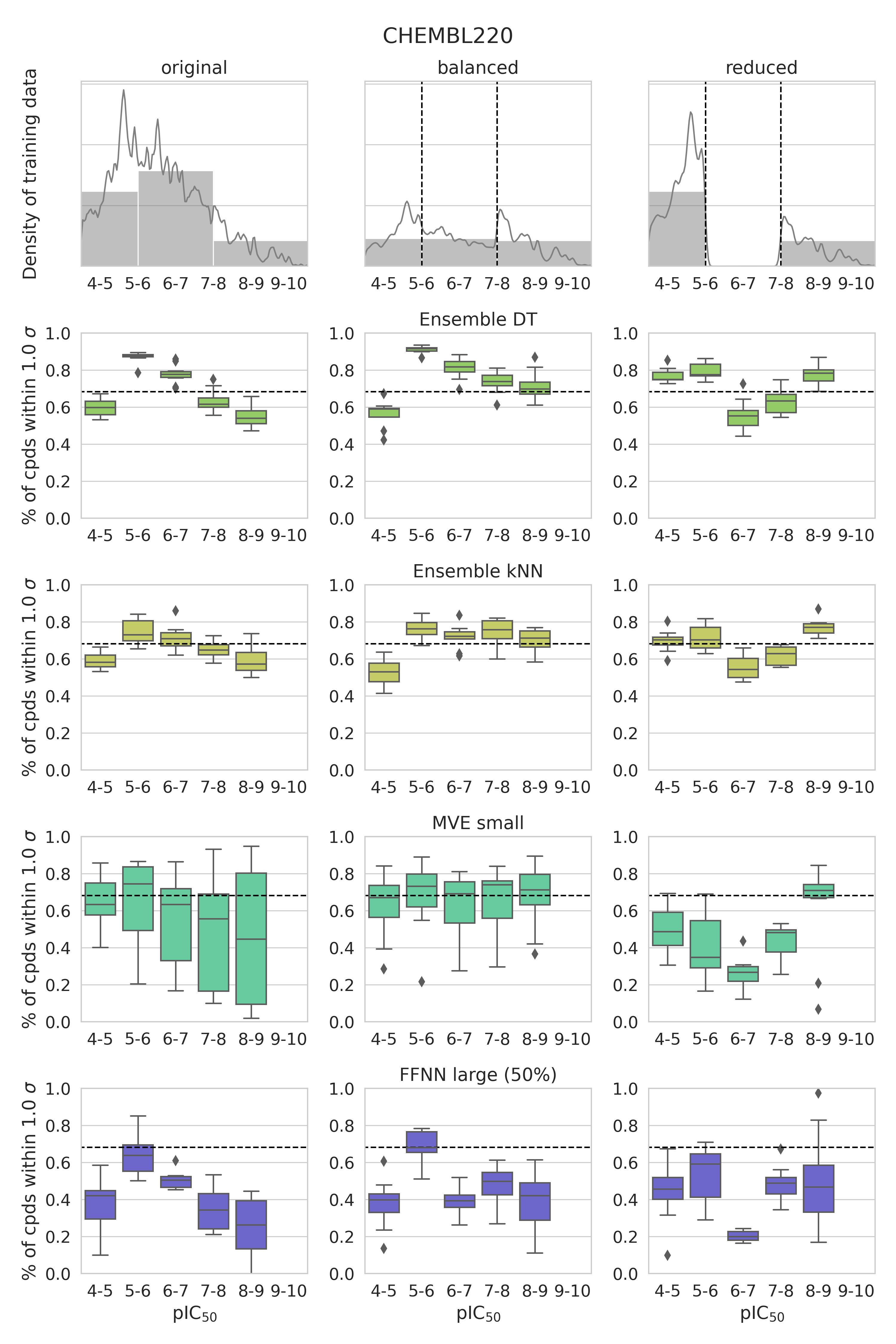


**Fig. S2b**


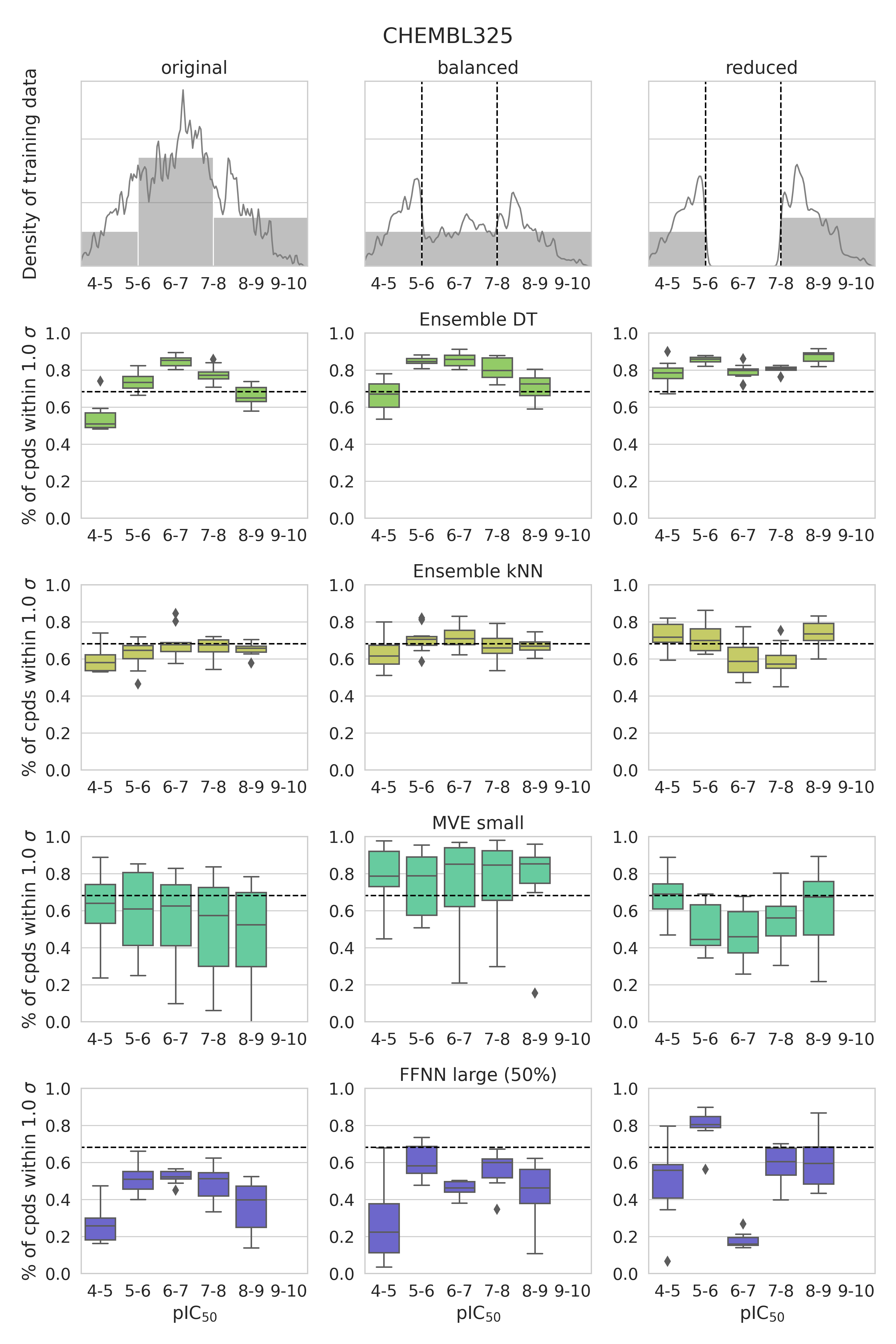


**Fig. S2c**


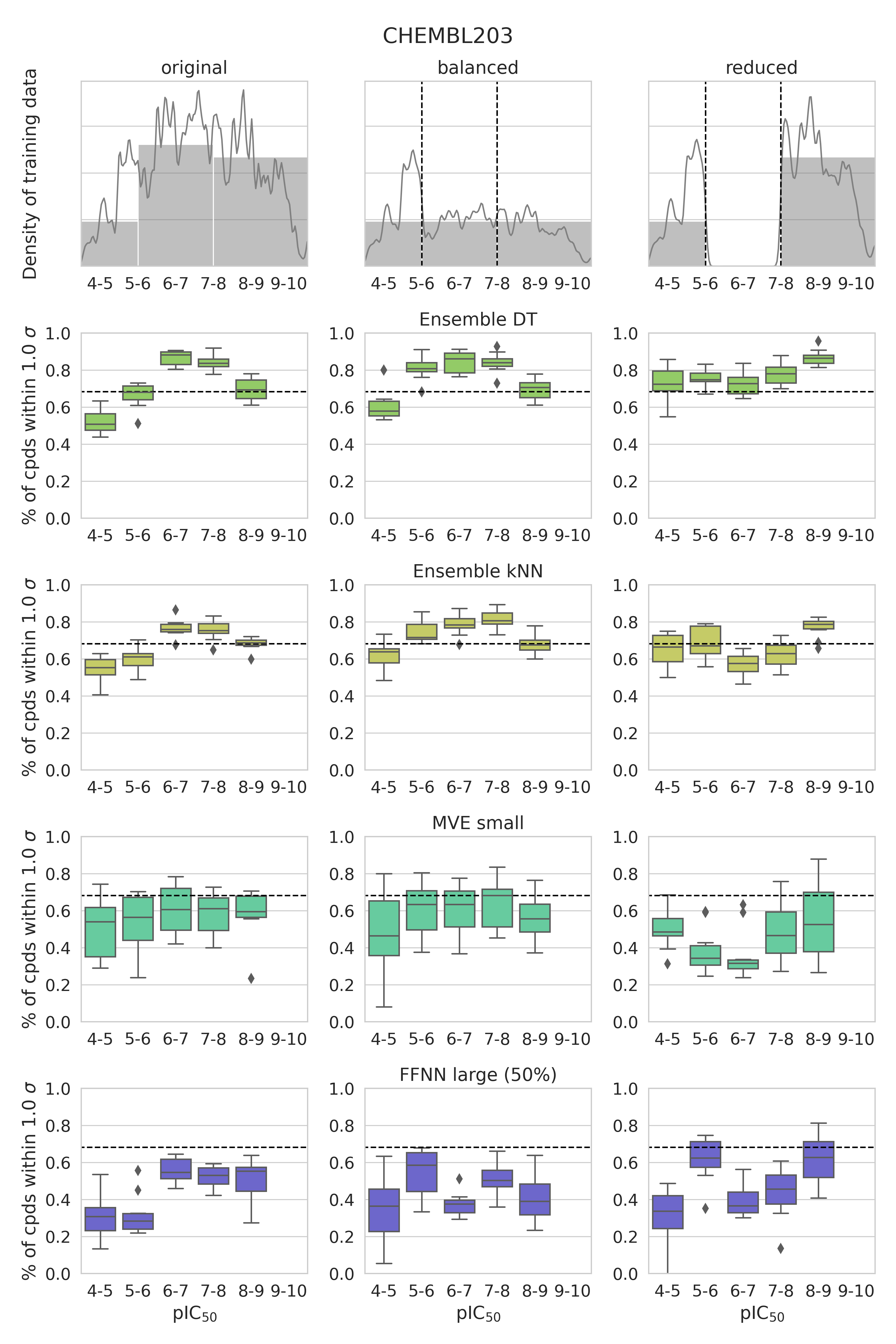

Supplement: Supplementary file 1 — Supplementary Figures. [file 41598_2024_57135_MOESM1_ESM.docx]
